# Factors associated with the initiation of laxative use in the same patients with schizophrenia over a 20‐year period: Retrospective cohort study

**DOI:** 10.1002/npr2.12378

**Published:** 2023-09-12

**Authors:** Yasushi Kawamata, Norio Sugawara, Taro Sasaki, Saaya Yokoyama, Hiroaki Okayasu, Masataka Shinozaki, Yoshitaka Takeuchi, Aoi Sato, Takaaki Ishikawa, Hazuki Komahashi‐Sasaki, Kensuke Miyazaki, Takashi Fukasawa, Hanako Furukori, Norio Yasui‐Furukori

**Affiliations:** ^1^ Department of Psychiatry, School of Medicine Dokkyo Medical University Tochigi Japan; ^2^ Department of Psychiatry Kikuchi Hospital Tochigi Japan; ^3^ Department of Psychiatry TMC Shimotsuga Tochigi Japan; ^4^ Department of Psychiatry Asahi Hospital Tochigi Japan; ^5^ Department of Psychiatry Aoki Hospital Tochigi Japan; ^6^ Department of Psychiatry Fudogaoka Hospital Saitama Japan; ^7^ Department of Psychiatry Takizawa Hospital Tochigi Japan; ^8^ Department of Psychiatry Okamotodai Hospital Tochigi Japan; ^9^ Department of Psychiatry Muroi Hospital Tochigi Japan; ^10^ Department of Psychiatry Saitama‐Konan Hospital Saitama Japan; ^11^ Department of Psychiatry Kanuma Hospital Tochigi Japan; ^12^ Department of Neuropsychiatry Hirosaki‐Aiseikai Hospital Aomori Japan; ^13^ Department of Psychiatry Seinan Hospital Aomori Japan; ^14^ Department of Neuropsychiatry Kuroichi‐Akebono Hospital Aomori Japan

**Keywords:** antipsychotics, constipation, guideline, laxative, mood stabilizer, outpatients

## Abstract

**Background:**

Constipation is a common adverse effect of antipsychotics, but little investigation has been conducted. We aimed to address the factors associated with the initiation of laxative use in the same patients with schizophrenia over a 20‐year period.

**Methods:**

We enrolled patients with schizophrenia attending each hospital (*n* = 14) from April 1, 2021, and retrospectively examined all prescriptions as of April 1, 2016, 2011, 2006, and 2001, every 5 years starting in 2021, for this population. 716 participants with complete data were included in the analysis. The Cochran *Q* test followed by Bonferroni correction and the Cochran–Armitage trend test were used to determine the differences and trends of the frequency of each laxative. Multivariate logistic regression analysis was performed to assess the factors on the initiation of laxative use over a 20‐year period.

**Results:**

Of the patients, 25.1% were treated with laxatives in 2001, and 34.1% were treated in 2021. The numbers of patients treated with any laxatives significantly differed over the 20‐year period, with a significant increasing trend. In all laxatives, the numbers of patients treated with magnesium oxide, lubiprostone and elobixibat differed with a significant increasing trend. Female sex, age, the total DZP equivalent dose, and the doses of levomepromazine maleate, olanzapine, quetiapine, zotepine, lithium, and carbamazepine in 2021 were significant factors associated with the initiation of laxative use over the 20‐year period.

**Conclusions:**

Careful monitoring is needed for patients treated with levomepromazine maleate, olanzapine, quetiapine and zotepine. Optimizing prescriptions according to treatment guidelines could reduce antipsychotic‐induced constipation.

## Abstract 2

Of the patients (*n* = 716), 25.1% were treated with laxatives in 2001, and 34.1% were treated in 2021. The number of patients treated with laxatives differed, with an increasing trend over the past 20 years. Female sex, age, total DZP equivalent dose, and doses of lithium and carbamazepine in 2021 were significantly associated with the initiation of laxative use. Doses of levomepromazine maleate, olanzapine, quetiapine and zotepine in 2021 were significantly associated with the initiation of laxative use.

## Graphical abstract

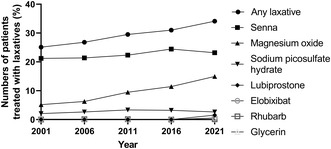

## 1 INTRODUCTION

Constipation is defined by bowel disturbances (reduced frequency of bowel movements, hard stools, excessive straining to defecate, a sense of anorectal blockage, anal digitation, and a sense of incomplete evacuation after defecation).^1^ The reported median prevalence of constipation was 16%.^2^ In the elderly population aged 60–101 years, constipation was more frequent, and the median prevalence rate was 33.5%.^2^ The reported risk factors for constipation are increasing age, female sex, lower socioeconomic status, lower parental education rates, less self‐reported physical activity and use of certain medications.^3^

Constipation is a well‐known adverse effect of antipsychotics, but there is a paucity of investigation regarding antipsychotic‐induced constipation. De Hert and colleagues^4^ investigated the prevalence of antipsychotic‐induced constipation over a period of 22 months and reported that 36.3% of patients required laxatives. One of the neurotransmitter mechanisms involved in antipsychotic‐induced constipation is the muscarinic anticholinergic effects of antipsychotics.^5^ Inappropriate treatment for antipsychotic‐induced constipation can lead to severe complications, including paralytic ileus, aspiration pneumonia and bowel perforation.^6^, ^7^ Because of the high case‐fatality rate of clozapine‐induced life‐threatening gastrointestinal hypomotility (15%–27.5%),^8^ most previous studies regarding antipsychotic‐induced constipation have focused on clozapine. Thus, little has been investigated regarding antipsychotic‐induced constipation not focused on clozapine. Recently, Lin and colleagues^9^, ^10^ investigated the factors associated with regular laxative use at discharge in schizophrenia patients treated with second‐generation antipsychotic (SGA) monotherapy. They reported that advanced age and higher antipsychotic or anticholinergic doses were associated with an increase in laxative use. Among SGAs, clozapine was associated with the highest rate of laxative use, followed by zotepine, quetiapine, olanzapine and risperidone.^9^

No study to date has examined the factors linked to antipsychotic‐induced constipation in a longitudinal manner within individual patients. The existing studies on this topic have primarily been cross‐sectional or descriptive in nature. Laxative use is a proxy major of antipsychotic‐induced constipation. Therefore, we aimed to address the factors associated with the initiation of laxative use in the same patients with schizophrenia over 20 years of age. 716 participants with complete data were included in the analysis.

## 2 METHODS

### 2.1 Participants

This study was initiated in April 2021 and was conducted in 14 psychiatric hospitals.^11^ Participants were selected based on the consecutive sampling method. To obtain an equivalent number of participants from each site, we set the number (*n* = 70) due to the feasibility. We enrolled up to 70 patients with schizophrenia attending each hospital from April 1, 2021, whose prescriptions could be traced over the past 20 years; for hospitals that did not reach 70 patients, all patients were enrolled. We excluded patients who had been off antipsychotic medication for more than 2 years in the past 20 years or who were untraceable because they had been treated at another hospital in the past 20 years. All patients included in this study were diagnosed with schizophrenia according to the Diagnostic and Statistical Manual of Mental Disorders, fifth edition, or the International Classification of Disease, tenth revision. Patients with clinically significant medical illnesses or active psychotic symptoms related to comorbid substance use disorders were excluded (e.g., disturbance of consciousness due to a stroke or alcohol withdrawal delirium). The medical records of the patients were reviewed to obtain all medications, including laxatives, and demographic data (age and sex). Starting in 2021, we conducted a retrospective analysis of prescription records for this population at 5‐year intervals, specifically for the years 2016, 2011, 2006, and 2001. A chlorpromazine equivalent for antipsychotics, biperiden equivalent for anticholinergic drugs, imipramine equivalent for antidepressants, diazepam (DZP) equivalent for anxiolytics and hypnotics were used for the purpose of comparison.^12^, ^13^

### 2.2 Statistical analysis

**FIGURE 1:**
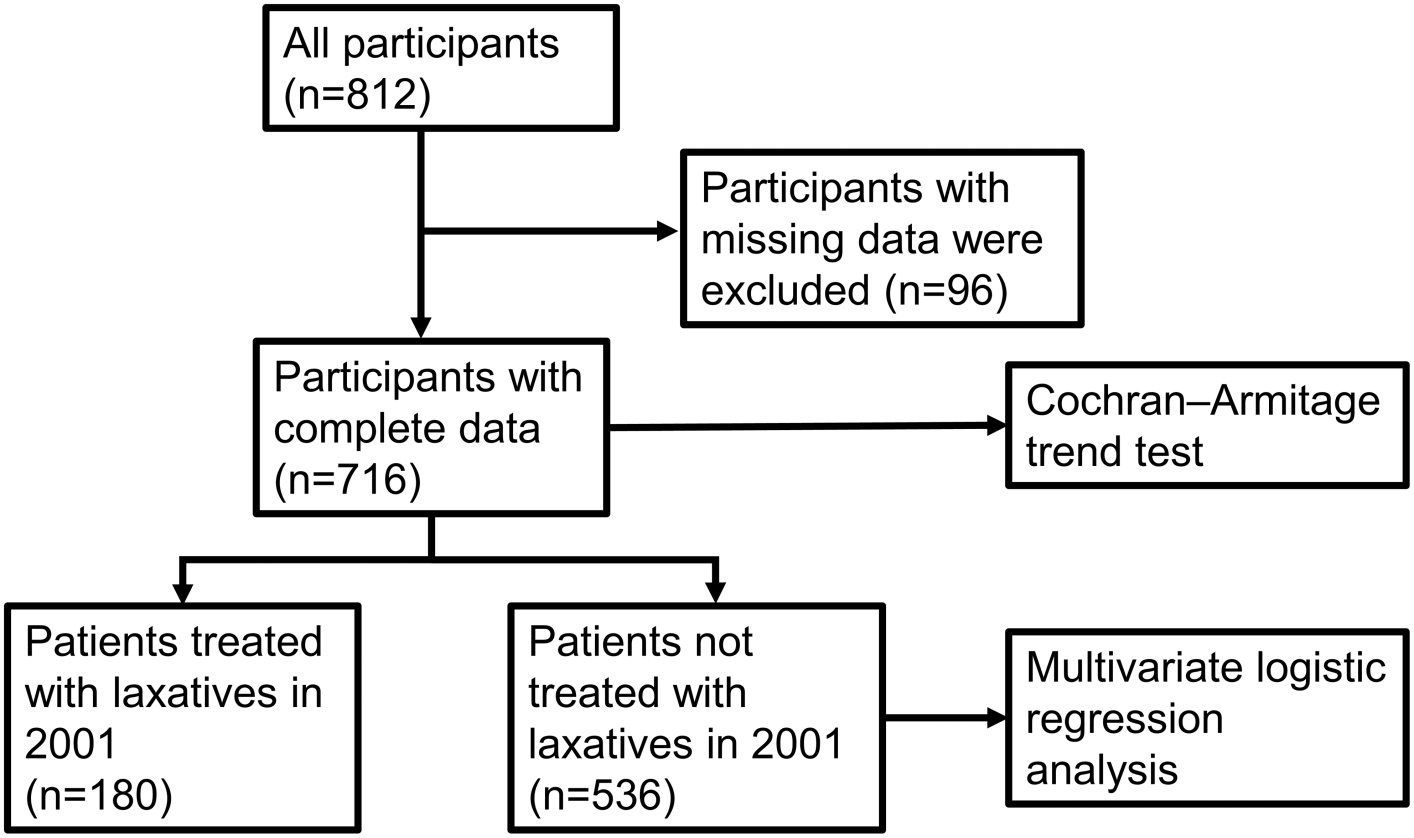
Procedure for statistical analysis.

The data are presented as the mean and standard deviation (SD) and number (%). A *p*‐value of <0.05 indicated statistical significance. The procedure for the statistical analysis of our research is shown in Figure 1. Data were obtained for 812 participants in total, but since there were 96 participants with missing data, 716 participants with complete data were included in the analysis. The Cochran *Q* test followed by Bonferroni correction and Cochran–Armitage trend test were used to determine the differences and trends of the frequency of each laxative among the five groups (*n* = 716). The paired *t*‐test was used to determine the differences between the biperiden equivalent of total anticholinergic drug between 2001 and 2021 (*n* = 716). For multivariate logistic regression analysis, patients treated with laxatives in 2001 (*n* = 180) were excluded, and patients not treated with laxatives in 2001 (*n* = 536) were included. Multivariate logistic regression analysis with a forward selection method was performed to assess the effects of age, sex, chlorpromazine equivalent of total antipsychotics, total first generation antipsychotics (FGA) and total SGA, biperiden equivalent of total anticholinergic drugs, imipramine equivalent of total antidepressants, diazepam equivalent of total anxiolytics and hypnotics, daily dose of each antipsychotic (risperidone, olanzapine, aripiprazole, quetiapine, blonanserin, zotepine, brexpiprazole, perospirone, asenapine maleate, clozapine, lurasidone, haloperidol, levomepromazine maleate, chlorpromazine, paliperidone, sulpiride, bromperidol, fluphenazine maleate, perphenazine maleate, propericiazine, clocapramine hydrochloride hydrate, mosapramine, pipamperone hydrochloride, nemonapride) and mood stabilizers (valproate, lithium, carbamazepine, lamotrigine) in 2021 and biperiden equivalent of total anticholinergic drugs in 2001 on the initiation of laxative use over 20 years (those who were not treated with laxatives in 2001 but treated in 2021 were scaled as 1, and the others were scaled as 0). The data were analyzed using SPSS statistics software for Windows version 27.0.0.0 (Japan IBM, Tokyo, Japan) and R for Windows Version 4.2.2 (The R Foundation for Statistical Computing, Vienna, Austria).

## 3 RESULTS

**FIGURE 2:**
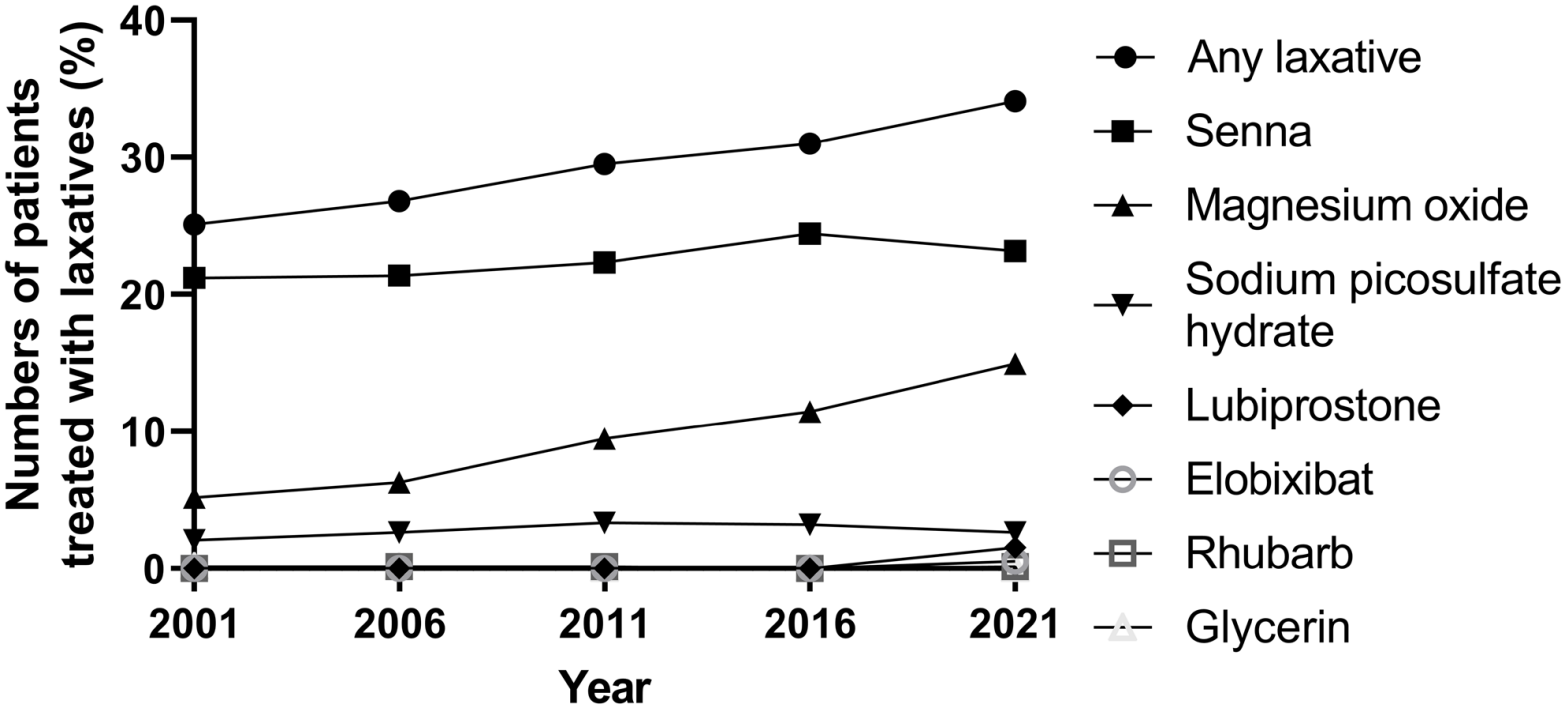
Increased number of patients treated with laxatives.

The clinical and demographic characteristics of the participants are listed in Table 1, and the numbers of the prescribed antipsychotics and mood stabilizers in 2021 are listed in Table S1. The frequencies of prescribed laxatives and the numbers of patients treated with laxatives over 20 years are shown in Table 2 and Figure 2. Laxatives prescribed in this study were magnesium oxide, senna, sodium picosulfate hydrate, lubiprostone, elobixibat, rhubarb, and glycerin (enema). The number of patients treated with any laxatives differed over the 20 years (Cochran *Q* = 44.3, df = 4, *p* < 0.001), with an increasing trend over the past 20 years (χ^2^ = 16.83, df = 1, *p* = 0.028). Bonferroni's post hoc analysis of the Cochran *Q* test revealed that the number of patients treated with any laxatives in 2021 was significantly larger than the number in 2001, 2006, and 2011 (*p* < 0.001, *p* < 0.001, *p* = 0.020, respectively). The number of patients treated with any laxatives in 2016 was significantly larger than the number in 2001 and 2006 (*p* = 0.001 and *p* < 0.049, respectively). The number of patients treated with any laxatives in 2011 was significantly larger than the number in 2001 (*p* = 0.037). The number of patients treated with magnesium oxide differed over the 20 years (Cochran *Q* = 97.4, df = 4, *p* < 0.001), with an increasing trend over the past 20 years (χ^2^ = 51.04, df = 1, *p* < 0.001). Bonferroni's post hoc analysis of the Cochran *Q* test revealed that the number of patients treated with magnesium oxide in 2021 was significantly larger than the number in 2001, 2006, 2011 and 2016 (*p* < 0.001, *p* < 0.001, *p* < 0.001, *p* = 0.027, respectively). The number of patients treated with magnesium oxide in 2016 was significantly larger than the number in 2001 and 2006 (*p* < 0.001 and *p* < 0.001, respectively). The number of patients treated with magnesium oxide in 2011 was significantly larger than the number in 2006 and 2001 (*p* = 0.041 and *p* = 0.001, respectively). The number of patients treated with senna did not differ over the 20 years (Cochran *Q* = 8.13, df = 4, *p* = 0.087), and did not show trend over the past 20 years (χ^2^ = 2.00, df = 1, *p* = 0.157). The number of patients treated with sodium picosulfate hydrate did not differ over the 20 years (Cochran *Q* = 5.53, df = 4, *p* = 0.237), and did not show trend over the past 20 years (χ^2^ = 0.74, df = 1, *p* = 0.389). The number of patients treated with lubiprostone differed over the 20 years (Cochran *Q* = 38.8, df = 4, *p* < 0.001), with an increasing trend over the past 20 years (χ^2^ = 22.06, df = 1, *p* = 0.006). Bonferroni's post hoc analysis of the Cochran *Q* test revealed that the number of patients treated with lubiprostone in 2021 was significantly larger than the number in 2001, 2006, 2011, and 2016 (*p* < 0.001, *p* < 0.001, *p* < 0.001, *p* < 0.001, respectively). The number of patients treated with elobixibat differed over the 20 years (Cochran *Q* = 16.0, df = 4, *p* = 0.003), with an increasing trend over the past 20 years (χ^2^ = 8.00, df = 1, *p* = 0.004). Bonferroni's post hoc analysis of the Cochran *Q* test revealed that the number of patients treated with elobixibat in 2021 was significantly larger than the number in 2001, 2006, 2011, and 2016 (*p* = 0.016, *p* = 0.016, *p* = 0.016, *p* = 0.016, respectively). The number of patients treated with rhubarb did not differ over the 20 years (Cochran *Q* = 4.00, df = 4, *p* = 0.406), and did not show a trend over the past 20 years (χ^2^ = 0.16, df = 1, *p* = 0.683). The number of patients treated with glycerin did not differ over the 20 years (Cochran *Q* = 4.00, df = 4, *p* = 0.406), and did not show a trend over the past 20 years (χ^2^ = 2.25, df = 1, *p* = 0.133). The biperiden equivalent of total anticholinergic drug in 2021 was smaller than that in 2001 (*t* = 16.0, df = 715, *p* < 0.001). Table 3 shows the factors associated with the initiation of laxative use over 20 years. With a multivariate logistic regression analysis with a forward selection method, female sex, age in 2021, total DZP equivalent dose in 2021, dose of levomepromazine maleate in 2021, dose of olanzapine in 2021, dose of quetiapine in 2021, dose of zotepine in 2021, dose of lithium in 2021, and dose of carbamazepine in 2021 were significantly associated with the initiation of laxative use over the 20‐year period.

**TABLE 1:** Clinical and demographic characteristics (*N* = 716).

Characteristics	2001	2006	2011	2016	2021
Age (years), mean (SD)	41.6 (11.5)	46.6 (11.5)	51.6 (11.5)	56.6 (11.5)	61.6 (11.5)
Female sex, *n* (%)	351 (49.0%)	351 (49.0%)	351 (49.0%)	351 (49.0%)	351 (49.0%)
Total antipsychotics^a^, *n*, mean (SD)	*n* = 696, 520 (523)	*n* = 707, 545 (535)	*n* = 707, 557 (490)	*n* = 711, 539 (489)	*n* = 701, 547 (480)
FGA, *n*, mean (SD)	*n* = 613, 433 (485)	*n* = 555, 363 (457)	*n* = 506, 297 (399)	*n* = 466, 267 (379)	*n* = 392, 230 (291)
SGA, *n*, mean (SD)	*n* = 207, 432 (271)	*n* = 352, 506 (372)	*n* = 440, 545 (382)	*n* = 468, 543 (390)	*n* = 505, 581 (443)
Anticholinergic drugs^b^, *n*, mean (SD)	*n* = 585, 3.9 (2.4)	*n* = 558, 3.3 (2.0)	*n* = 507, 3.2 (2.0)	*n* = 464, 3.0 (1.8)	*n* = 411, 2.9 (1.7)
Antidepressants^c^, *n*, mean (SD)	*n* = 52, 69 (60)	*n* = 56, 68 (46)	*n* = 59, 72 (52)	*n* = 54, 74 (54)	*n* = 59, 73 (56)
Anxiolytics and hypnotics^d^, *n*, mean (SD)	*n* = 509, 19 (17)	*n* = 501, 19 (19)	*n* = 512, 18 (17)	*n* = 487, 16 (14)	*n* = 473, 14 (12)
Mood stabilizers and antiepileptics, (*n*, %)	137 (19.1%)	167 (23.3%)	184 (25.7%)	175 (24.4%)	157 (21.9%)

*Note*: Values are the mean and standard deviation (SD) or number (%).

Abbreviations: FGA, First‐generation antipsychotics; SGA, Second generation antipsychotics.

^a^ Chlorpromazine equivalent (mg/day).

^b^ Biperiden equivalent (mg/day).

^c^ Imipramine equivalent (mg/day).

^d^ Diazepam equivalent (mg/day).

**TABLE 2:** The frequencies of prescribed laxatives and the numbers of patients treated with laxatives in the same patients with schizophrenia over 20 years.

Year	2001	2006	2011	2016	2021	Trends
Laxatives						
Magnesium oxide	37	45	68	82	107	χ^2^ = 51.04, df = 1, *p* < 0.001
Senna	152	153	160	175	166	χ^2^ = 2.00, df = 1, *p* = 0.157
Sodium picosulfate hydrate	15	19	24	23	19	χ^2^ = 0.74, df = 1, *p* = 0.389
Lubiprostone	0	0	0	0	11	χ^2^ = 22.06, df = 1, *p* = 0.006
Elobixibat	0	0	0	0	4	χ^2^ = 8.00, df = 1, *p* = 0.004
Rhubarb	0	1	1	0	1	χ^2^ = 0.16, df = 1, *p* = 0.683
Glycerin	1	1	0	0	0	χ^2^ = 2.25, df = 1, *p* = 0.133
Numbers of patients treated with laxatives^a^ (%)	180/716 (25.1%)	192/716 (26.8%)	211/716 (29.5%)	222/716 (31.0%)	244/716 (34.1%)	χ^2^ = 16.83, df = 1, *p* = 0.028

^a^ Coadministration of laxatives was not counted in duplicate.

**TABLE 3:** Factors associated with the initiation of laxative use in the same patients with schizophrenia over 20 years.

	B	Standard error	Wald value	*p*‐Value	Odds ratio
Female sex	0.590	0.229	6.658	0.010	1.804 (1.152–2.825)
Age	0.026	0.010	6.062	0.014	1.026 (1.005–1.047)
Total DZP equivalent dose	0.019	0.009	4.771	0.029	1.019 (1.002–1.036)
Levomepromazine maleate	0.011	0.004	8.336	0.004	1.011 (1.003–1.018)
Olanzapine	0.054	0.020	7.456	0.006	1.055 (1.015–1.097)
Quetiapine	0.004	0.001	10.634	0.001	1.004 (1.002–1.007)
Zotepine	0.010	0.003	9.719	0.002	1.010 (1.004–1.016)
Lithium	0.002	0.001	5.153	0.023	1.002 (1.000–1.003)
Carbamazepine	0.003	0.001	5.338	0.021	1.003 (1.000–1.005)

Abbreviation: DZP, diazepam.

## 4 DISCUSSION

To the best of our knowledge, this is the first study to investigate the factors associated with the initiation of laxative use in the same patients with schizophrenia over a 20‐year period. Of the patients in our survey, 25.1% were treated with laxatives in 2001, and 34.1% were treated in 2021. Female sex, age in 2021, total DZP equivalent dose in 2021, dose of levomepromazine maleate in 2021, dose of olanzapine in 2021, dose of quetiapine in 2021, dose of zotepine in 2021, dose of lithium in 2021, and dose of carbamazepine in 2021 were significant factors associated with the initiation of laxative use over a 20‐year period.

The prevalence of laxative use in this survey (25.1%–34.1%) was consistent with the previous study by De Hert and their colleagues^4^ (36.3%) and Lin and their colleagues^9^ (22.1%–33.7%) but smaller than that reported by Koizumi and their colleagues^14^ (68.0%). Because antipsychotic‐induced constipation is more common in inpatient settings than in outpatient and mixed settings,^15^ the prevalence of laxative use in our survey differed from that in a previous study by Koizumi and their colleagues,^14^ which involved inpatient settings. Compared to the previous study which reported that prevalence rate of constipation in elderly individuals was 33.5%,^2^ the frequency of laxative use in patients with schizophrenia in this study was almost the same and not high. It might be due to oversight of constipation because patients with schizophrenia were less aware of constipation and less reported its presence to their psychiatrists.^14^ Our results showed an increasing trend of laxative use over the past 20 years, and age in 2021 was a significant factor associated with the initiation of laxative use, which is in line with a previous study showing that increasing age is a risk factor for constipation.^3^ Female sex was a significant factor associated with the initiation of laxative use. This is natural since female sex is known as a risk factor for constipation.^3^ The total DZP equivalent dose in 2021 was a significant factor associated with the initiation of laxative use. This is in line with the reported adverse events of benzodiazepine, which are due to the anticholinergic effect of benzodiazepine.^16^ Among the antipsychotics used in our study, daily doses of levomepromazine maleate, olanzapine, quetiapine and zotepine in 2021 were significant factors associated with the initiation of laxative use. This is in line with a previous study showing that zotepine, quetiapine and olanzapine had the highest rates of laxative use in SGA, followed by clozapine,^9^ and levomepromazine maleate was associated with severe constipation.^17^ In our survey, clozapine was not a significant factor associated with the initiation of laxative use. This is not consistent with previous studies that reported that clozapine‐induced constipation is most prevalent and severe.^5^, ^9^, ^10^, ^15^ This might be due to a lack of statistical power because only two participants were treated with clozapine in our survey in 2021. Daily doses of lithium and carbamazepine in 2021 were significant factors associated with the initiation of laxative use. This is consistent with a previous study that reported that lithium and carbamazepine were the medications associated with constipation.^3^ The biperiden equivalent of total anticholinergic drug in 2021 was smaller than that in 2001, as we previously reported.^11^ There was no significance between the initiation of laxative use and the daily dose of anticholinergic drugs in 2021 or 2001. This is not consistent with a previous study by Lin and their colleagues who reported that a higher dose of anticholinergic drugs was associated with increasing laxative use.^9^ However, in the elderly population (average age 66.3), they reported that antiparkinsonian agent use was not associated with laxative use,^10^ which is in line with our results.

Osmotic laxatives such as magnesium oxide are recommended as a first‐line treatment by several recent guidelines for chronic idiopathic constipation.^18^, ^19^ The number of patients treated with magnesium oxide showed increasing trend over the past 20 years, which is in line with these guidelines. Stimulant laxatives such as senna, sodium picosulfate hydrate and rhubarb are recommended as second‐line treatment by several guidelines.^18^, ^19^, ^20^, ^21^ The number of patients treated with senna, sodium picosulfate hydrate or rhubarb showed no increasing trend over the past 20 years, which is in line with these guidelines. We did not assess the dose of laxatives and their acclimatizing effects in this study. Careful consideration of this point is needed. Lubiprostone and elobixibat, which are newer pharmacological agents, showed increasing trend over the past 20 years. These agents are not recommended in the guidelines, because they are not yet available in most countries.^18^, ^19^, ^20^, ^21^ The number of patients treated with glycerin (enema) showed no increasing trend over the past 20 years. Several guidelines state the efficacy of enemas,^18^, ^20^, ^21^ but mention that a trial is probably justified in patients in whom all other measures have failed.^20^

To reduce antipsychotic‐induced constipation, one possible solution is to optimize the prescription according to treatment guidelines. Previously in this survey, we reported a slow but steady substitution of SGAs for FGAs over time.^11^ Since many guidelines recommend that monotherapy with SGAs is the first‐line treatment for schizophrenia,^22^, ^23^, ^24^ polytherapy with benzodiazepine and mood stabilizers, including lithium and carbamazepine, should be minimized. In Japan, the Effectiveness of Guidelines for Dissemination and Education (EGUIDE) psychiatric treatment project, which aims to disseminate the guidelines that present the treatments for schizophrenia and major depressive disorder via education programs for psychiatrists, was reported to be effective.^25^, ^26^, ^27^ In addition, to easily visualize the degree to which current prescriptions conform to the guidelines for the pharmacological treatment of schizophrenia,^24^ the individual fitness score (IFS) is an effective tool, in which points vary in range from 0 to 100.^28^ To prevent severe complications due to antipsychotic‐induced constipation, careful monitoring is needed for patients treated with high‐risk antipsychotics for constipation such as levomepromazine maleate, olanzapine, quetiapine, zotepine and clozapine. In cases of severe constipation, decreasing the dose of antipsychotics or switching to low‐risk antipsychotics should be considered.

There are several limitations of this study. First, we adopted laxative use as a proxy measure for constipation. Some participants with constipation may not have been treated with laxatives in our study because patients with schizophrenia are less aware of constipation and less frequently report its presence to their psychiatrists.^14^ Second, our study did not assess the laxatives prescribed in other hospitals because our study was conducted in outpatient settings. However, it is also a strength because most of the patients with schizophrenia were outpatients, and previous studies were inpatient settings.^4^, ^9^, ^10^, ^14^ Third, there may be sampling bias because the participants were not randomized, and those who continued to visit the same hospital over 20 years were selected. Fourth, the coadministration of antipsychotics was not assessed. Fifth, the coadministration or dose of laxatives was not assessed. Sixth, several potential confounding factors, such as comorbidities, activities of daily living, fiber/fluid intake, duration of illness, and severity of illness, were not assessed in our study. Finally, the odds ratios for the factors associated with the initiation of laxative use, except for gender, are significant but very small. Careful consideration of this point is needed.

## 5 CONCLUSION

Of the patients, 25.1% were treated with laxatives in 2001, and 34.1% were treated in 2021. Female sex, age in 2021, the total DZP equivalent dose in 2021, the dose of levomepromazine maleate in 2021, the dose of olanzapine in 2021, the dose of quetiapine in 2021, the dose of zotepine in 2021, the dose of lithium in 2021, and the dose of carbamazepine in 2021 were shown to be associated with the initiation of laxative use over the 20‐year period. Careful monitoring is needed for patients treated with high‐risk antipsychotics for constipation such as zotepine, quetiapine, olanzapine and levomepromazine maleate. Optimizing prescriptions according to treatment guidelines could reduce antipsychotic‐induced constipation. Further studies on the promotion of treatment guidelines are needed.

## AUTHOR CONTRIBUTIONS

All authors contributed to the design of this study. Dr. Kawamata analyzed the data and wrote the first draft of the manuscript. All authors have contributed to and approved the final manuscript.

## FUNDING INFORMATION

The authors received no specific funding for this work.

## CONFLICT OF INTEREST STATEMENT

Yasushi Kawamata has received speaker's honoraria from Eisai and Sumitomo Pharma Co., Ltd., outside the submitted work. Kensuke Miyazaki reports personal fees from Sumitomo Pharma Co., Ltd., outside the submitted work. The remaining authors report no other conflicts of interest in this work.

## ETHICS STATEMENT

Approval of the research protocol by an Institutional Reviewer Board: The data collection protocol for this study was approved by the Ethics Committee of the Dokkyo Medical University Hospital (Ref. R‐54‐3J). This protocol was conducted in accordance with the principles of the Declaration of Helsinki and the Japanese Ethical Guidelines for Medical and Health Research Involving Human Subjects.

Informed consent: Since this was a retrospective medical record survey, it was exempted from the requirement for informed consent; however, we released information on this research so that patients were free to opt‐out.

Registry and the registration no. of the study/trial: N/A.

Animal Studies: N/A.

## Supporting information


Table S1.

## Data Availability

The Ethics Committee of the Dokkyo Medical University Hospital has set restrictions on data sharing because the data contain potentially identifying or sensitive patient information. Please contact the institutional review board of the ethics committee of the Dokkyo Medical University Hospital for data requests. Upon request, the ethics committee will decide whether to share the data.

## References

[1] Bharucha AE , Wald A . Chronic constipation. Mayo Clin Proc. 2019;94(11):2340–2357. 31054770 10.1016/j.mayocp.2019.01.031 PMC6829047

[2] Mugie SM , Benninga MA , Di Lorenzo C . Epidemiology of constipation in children and adults: a systematic review. Best Pract Res Clin Gastroenterol. 2011;25(1):3–18. 21382575 10.1016/j.bpg.2010.12.010

[3] Bharucha AE , Pemberton JH , Locke GR 3rd. American Gastroenterological Association technical review on constipation. Gastroenterology. 2013;144(1):218–238. 23261065 10.1053/j.gastro.2012.10.028 PMC3531555

[4] De Hert M , Dockx L , Bernagie C , Peuskens B , Sweers K , Leucht S , et al. Prevalence and severity of antipsychotic related constipation in patients with schizophrenia: a retrospective descriptive study. BMC Gastroenterol. 2011;11:17. 21385443 10.1186/1471-230X-11-17 PMC3062582

[5] Xu Y , Amdanee N , Zhang X . Antipsychotic‐induced constipation: a review of the pathogenesis, clinical diagnosis, and treatment. CNS Drugs. 2021;35(12):1265–1274. 34427901 10.1007/s40263-021-00859-0

[6] Freudenreich O , Goff DC . Colon perforation and peritonitis associated with clozapine. J Clin Psychiatry. 2000;61(12):950–951. 11206605 10.4088/jcp.v61n1210e

[7] Chen HK , Hsieh CJ . Risk of gastrointestinal Hypomotility in schizophrenia and schizoaffective disorder treated with antipsychotics: a retrospective cohort study. Schizophr Res. 2018;195:237–244. 29107449 10.1016/j.schres.2017.10.024

[8] Cohen D , Bogers JP , van Dijk D , Bakker B , Schulte PF . Beyond white blood cell monitoring: screening in the initial phase of clozapine therapy. J Clin Psychiatry. 2012;73(10):1307–1312. 23140648 10.4088/JCP.11r06977

[9] Lin CH , Chan HY , Hsu CC , Chen FC . Factors associated with laxative use in schizophrenia patients treated with second‐generation antipsychotics. Eur Neuropsychopharmacol. 2021;43:139–146. 33419642 10.1016/j.euroneuro.2020.12.008

[10] Lin CH , Lin HY , Lin TC , Chan HY , Chen JJ . The relation between second‐generation antipsychotics and laxative use in elderly patients with schizophrenia. Psychogeriatrics. 2022;22(5):718–727. 35810468 10.1111/psyg.12875

[11] Yasui‐Furukori N , Kawamata Y , Sasaki T , Yokoyama S , Okayasu H , Shinozaki M , et al. Prescribing trends for the same patients with schizophrenia over 20 years. Neuropsychiatr Dis Treat. 2023;19:921–928. 37089914 10.2147/NDT.S390482 PMC10120815

[12] Inada T , Inagaki A . Psychotropic dose equivalence in Japan. Psychiatry Clin Neurosci. 2015;69(8):440–447. 25601291 10.1111/pcn.12275

[13] Inagaki A , Inada T . Dose equivalence of psychotropic drugs(part 28)dose equivalence of novel antipsychotics: Brexpiprazole. Japanese J Clin Psychopharmacol. 2022;25(1):91–98.

[14] Koizumi T , Uchida H , Suzuki T , Sakurai H , Tsunoda K , Nishimoto M , et al. Oversight of constipation in inpatients with schizophrenia: a cross‐sectional study. Gen Hosp Psychiatry. 2013;35(6):649–652. 23871089 10.1016/j.genhosppsych.2013.06.007

[15] Shirazi A , Stubbs B , Gomez L , Moore S , Gaughran F , Flanagan RJ , et al. Prevalence and predictors of clozapine‐associated constipation: a systematic review and meta‐analysis. Int J Mol Sci. 2016;17(6):1–15. 10.3390/ijms17060863 PMC4926397 27271593

[16] Bruno E , Nicoletti A , Quattrocchi G , Filippini G , Zappia M , Colosimo C . Alprazolam for essential tremor. Cochrane Database Syst Rev. 2015;2015(12):Cd009681. 26638213 10.1002/14651858.CD009681.pub2 PMC7387361

[17] Bulot V , Lemogne C , Nebot N , Blondon H , Roux P . Systematic prevention of severe constipation induced by antipsychotic agents: a quasi‐experimental study. Eur Neuropsychopharmacol. 2016;26(10):1690–1691. 27546374 10.1016/j.euroneuro.2016.08.004

[18] Shin JE , Jung HK , Lee TH , Jo Y , Lee H , Song KH , et al. Guidelines for the diagnosis and treatment of chronic functional constipation in Korea, 2015 revised edition. J Neurogastroenterol Motil. 2016;22(3):383–411. 27226437 10.5056/jnm15185 PMC4930295

[19] Vitton V , Damon H , Benezech A , Bouchard D , Brardjanian S , Brochard C , et al. Clinical practice guidelines from the French National Society of Coloproctology in treating chronic constipation. Eur J Gastroenterol Hepatol. 2018;30(4):357–363. 29406436 10.1097/MEG.0000000000001080

[20] Serra J , Pohl D , Azpiroz F , Chiarioni G , Ducrotté P , Gourcerol G , et al. European society of neurogastroenterology and motility guidelines on functional constipation in adults. Neurogastroenterol Motil. 2020;32(2):e13762. 31756783 10.1111/nmo.13762

[21] Serra J , Mascort‐Roca J , Marzo‐Castillejo M , Aros SD , Ferrándiz Santos J , Rey Diaz Rubio E , et al. Clinical practice guidelines for the management of constipation in adults. Part 2: diagnosis and treatment. Gastroenterol Hepatol. 2017;40(4):303–316. 27045854 10.1016/j.gastrohep.2016.02.007

[22] Keepers GA , Fochtmann LJ , Anzia JM , Benjamin S , Lyness JM , Mojtabai R , et al. The American Psychiatric Association practice guideline for the treatment of patients with schizophrenia. Am J Psychiatry. 2020;177(9):868–872. 32867516 10.1176/appi.ajp.2020.177901

[23] Buchanan RW , Kreyenbuhl J , Kelly DL , Noel JM , Boggs DL , Fischer BA , et al. The 2009 schizophrenia PORT psychopharmacological treatment recommendations and summary statements. Schizophr Bull. 2010;36(1):71–93. 19955390 10.1093/schbul/sbp116 PMC2800144

[24] Japanese Society of Neuropsychopharmacology . Guideline for pharmacological therapy of schizophrenia. Neuropsychopharmacol Rep. 2021;41(3):266–324. 34390232 10.1002/npr2.12193 PMC8411321

[25] Hashimoto N , Yasui‐Furukori N , Hasegawa N , Ishikawa S , Numata S , Hori H , et al. Characteristics of discharge prescriptions for patients with schizophrenia or major depressive disorder: real‐world evidence from the effectiveness of guidelines for dissemination and education (EGUIDE) psychiatric treatment project. Asian J Psychiatr. 2021;63:102744. 34325252 10.1016/j.ajp.2021.102744

[26] Takaesu Y , Watanabe K , Numata S , Iwata M , Kudo N , Oishi S , et al. Improvement of psychiatrists' clinical knowledge of the treatment guidelines for schizophrenia and major depressive disorders using the 'Effectiveness of guidelines for dissemination and education in psychiatric treatment (EGUIDE)' project: a nationwide dissemination, education, and evaluation study. Psychiatry Clin Neurosci. 2019;73(10):642–648. 31437336 10.1111/pcn.12911 PMC6852015

[27] Yamada H , Motoyama M , Hasegawa N , Miura K , Matsumoto J , Ohi K , et al. A dissemination and education programme to improve the clinical behaviours of psychiatrists in accordance with treatment guidelines for schizophrenia and major depressive disorders: the effectiveness of guidelines for dissemination and education in psychiatric treatment (EGUIDE) project. BJPsych Open. 2022;8(3):e83. 35446248 10.1192/bjo.2022.44 PMC9059732

[28] Inada K , Fukumoto K , Hasegawa N , Yasuda Y , Yamada H , Hori H , et al. Development of individual fitness score for conformity of prescriptions to the "guidelines for pharmacological therapy of schizophrenia". Neuropsychopharmacol Rep. 2022;42(4):502–509. 36254805 10.1002/npr2.12293 PMC9773743

